# Case Report: Successful management of acute kidney injury following interventional heartworm extraction in a dog with caval syndrome

**DOI:** 10.3389/fvets.2025.1620928

**Published:** 2025-07-31

**Authors:** Jaehan Jun, Jaeyeon Yoon, Joohyun Jung

**Affiliations:** ^1^Ilsan Animal Medical Center, Goyang, Republic of Korea; ^2^Veterinary Emergency Medicine, Department of Veterinary Science, College of Veterinary Medicine, Seoul National University, Seoul, Republic of Korea

**Keywords:** acute kidney injury, caval syndrome, hemodialysis, interventional heartworm extraction, prolonged intermittent renal replacement therapy

## Abstract

A 12-year-old, 7 kg, castrated male, mixed-breed dog presented with lethargy, vomiting, and hemoglobinuria. Heartworm infection was diagnosed through a positive Dirofilaria immitis antigen test, thoracic radiography, and echocardiography, which revealed an extensive worm burden extending into the right atrium, right ventricle, main pulmonary artery, and caudal vena cava, indicative of caval syndrome. Interventional heartworm extraction was successfully performed via jugular venotomy, based on clinical and imaging assessments. However, within 48 h postoperatively, the dog developed acute kidney injury (AKI) characterized by oliguria, pleural effusion, and significantly elevated kidney biomarkers. Despite aggressive medical management, kidney function deteriorated, prompting two sessions of prolonged intermittent renal replacement therapy (PIRRT). After PIRRT, the dog exhibited marked clinical and biochemical improvements. Continuous follow-up demonstrated a progressive recovery of kidney function, which was supported by declining serum creatinine, blood urea nitrogen, and urinary cystatin B levels. Knowingly, this is the first documented report in the veterinary literature describing AKI secondary to caval syndrome following interventional heartworm removal in a dog that was successfully managed with PIRRT. This case underscores the necessity for early identification and management of kidney complications after heartworm extraction and highlights hemodialysis as an effective therapeutic modality for severe AKI associated with caval syndrome.

## Introduction

Caval syndrome is a life-threatening complication of heartworm disease, characterized by the intravascular migration of Dirofilaria immitis into the right atrium, right ventricle, and vena cava (1). This migration results in severe mechanical obstruction of the venous return and intravascular hemolysis, typically presenting as lethargy, anemia, and hemoglobinuria. Without timely intervention, the prognosis is poor (1–3). Transvenous heartworm extraction is the standard therapeutic approach for dogs with caval syndrome, and early recognition and prompt surgical intervention are critical for favorable outcomes (2–4). Although cardiovascular and hepatic complications of caval syndrome, including right-sided heart failure, pulmonary hypertension, and hepatic congestion, have been extensively documented (1, 5–12), kidney involvement remains underreported despite its clinical significance. Acute kidney injury (AKI) following heartworm extraction has been infrequently described in veterinary literature, and reports of cases necessitating kidney replacement therapy are especially rare. Here, we describe a case of caval syndrome in a dog that developed AKI following transvenous heartworm extraction and was successfully managed with prolonged intermittent renal replacement therapy (PIRRT). The potential mechanisms contributing to AKI are discussed, along with relevant therapeutic considerations regarding extracorporeal kidney support.

## Case description

**Figure 1:**
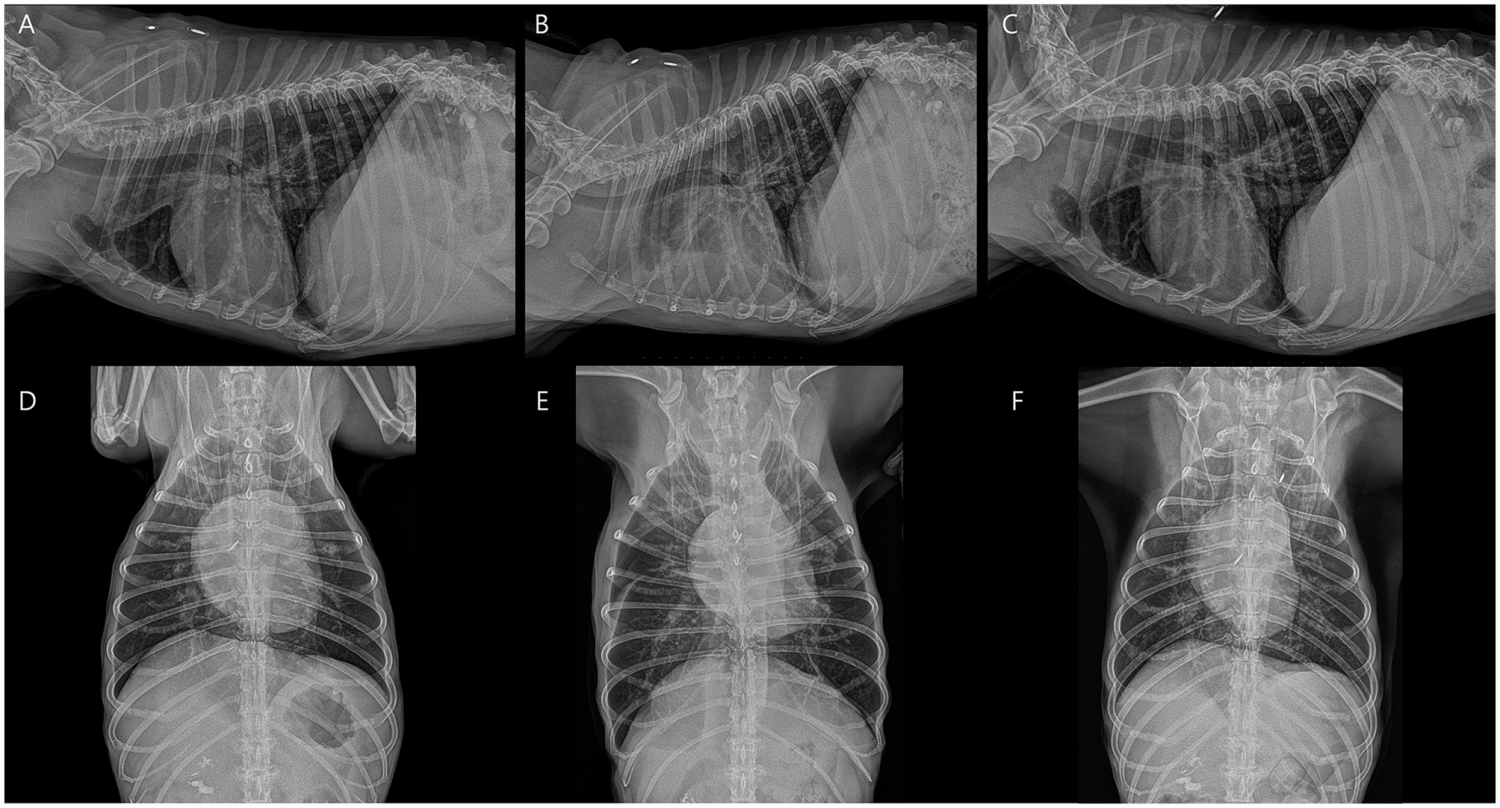
Serial thoracic radiographs illustrate cardiopulmonary changes associated with heartworm extraction and kidney failure. **(A, D)** Before heartworm extraction, radiographs reveal mild right side cardiomegaly with interstitial pulmonary infiltrates. **(B, E)** After heartworm extraction, acute kidney injury has developed, accompanied by pleural effusion. **(C, F)** Following heartworm extraction and resolution of kidney failure, cardiac and vascular sizes have decreased, with notably improved pleural effusion.

**Figure 2:**
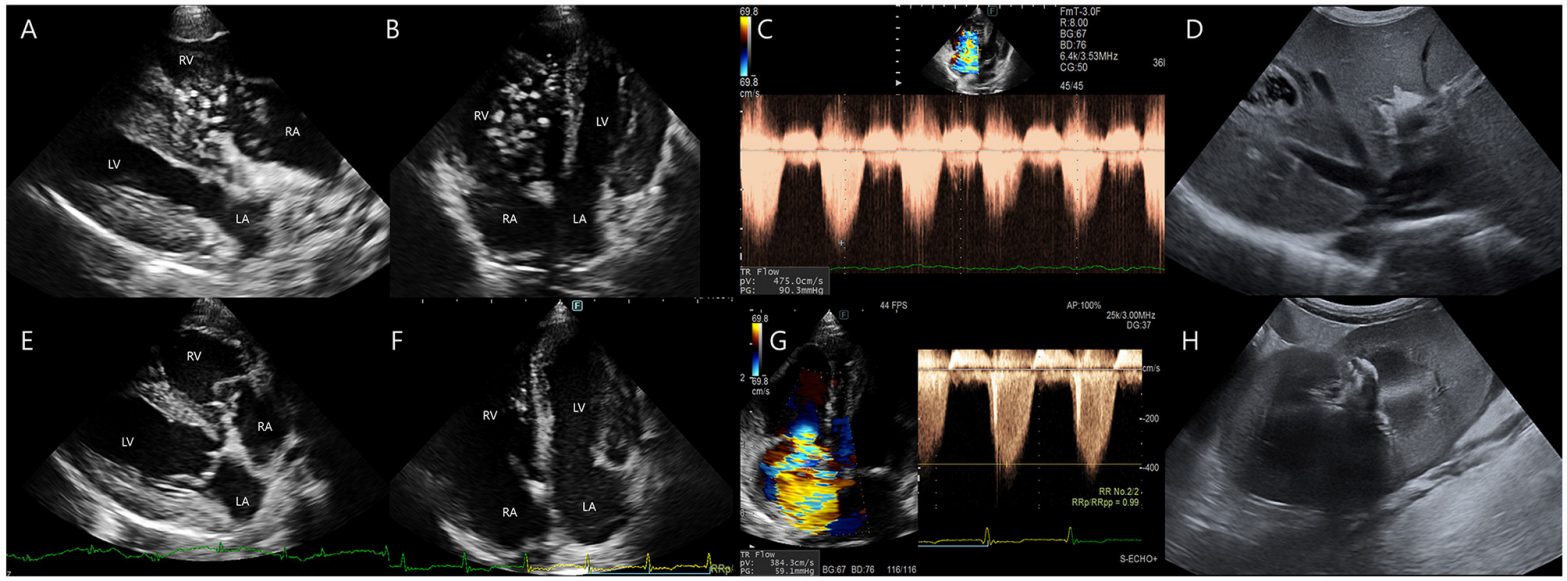
Transthoracic echocardiographic and abdominal ultrasonographic findings before and after heartworm extraction. **(A–D)** Pre-procedural echocardiographic and abdominal ultrasonographic images. **(A, B)** Multiple adult *Dirofilaria immitis* are noted within the dilated right ventricle and right atrium, exhibiting active movement between chambers. **(C)** Tricuspid regurgitation (TR) velocity is measured at 4.75 m/s, corresponding to a pressure gradient of 90.35 mmHg. **(D)** Heartworms are observed extending into the caudal vena cava. **(E–H)** Postoperative echocardiographic and abdominal ultrasonographic images. **(E, F)** Most intracardiac heartworms have been successfully removed. **(G)** TR velocity has decreased to 3.84 m/s (pressure gradient 59.1 mmHg). **(H)** Small amounts of subcapsular or perirenal fluid accumulation is noted 2 days after heartworm extraction.

**Figure 3:**
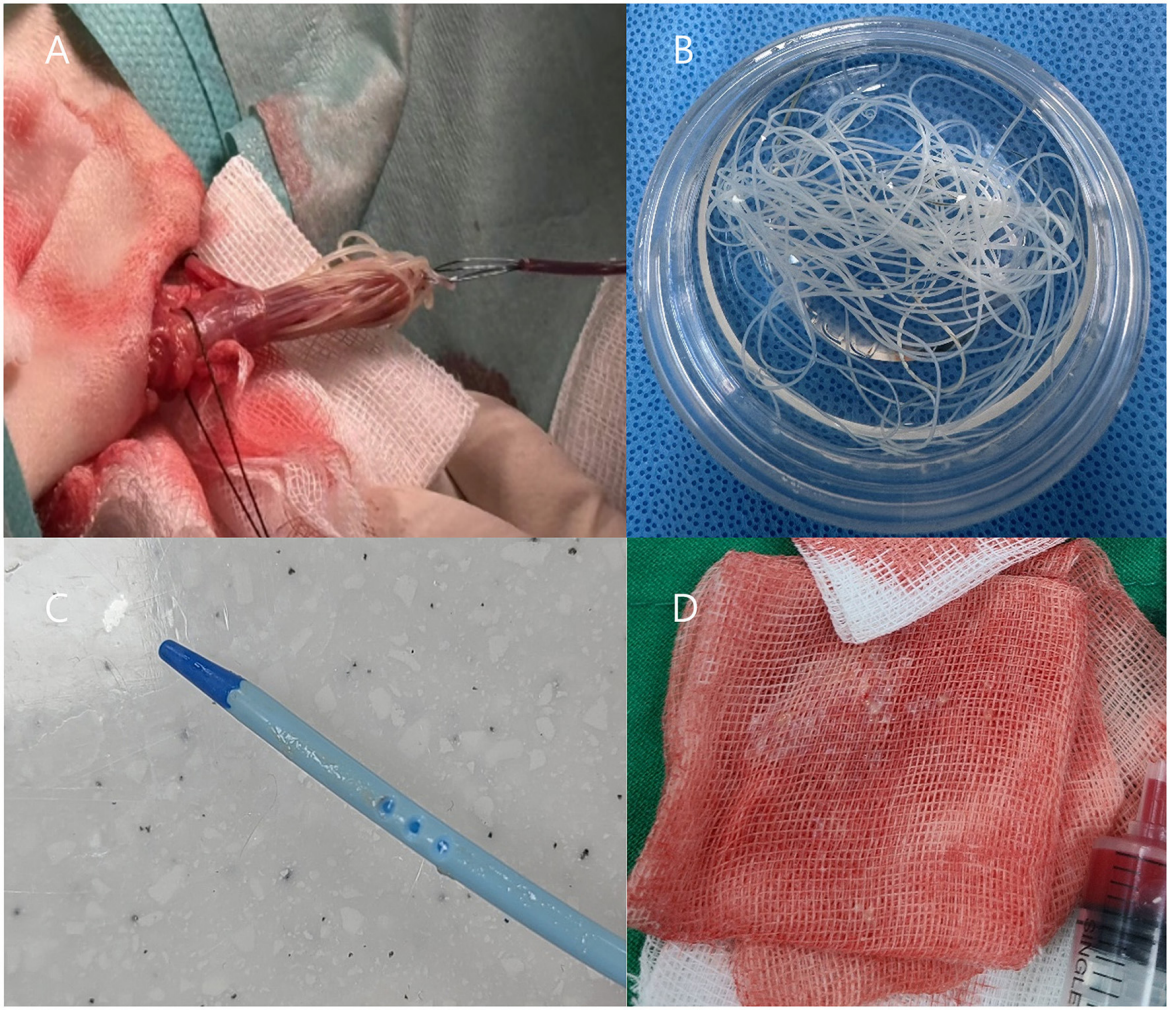
**(A, B)** Transvenous heartworm extraction via jugular venotomy, demonstrating adult *Dirofilaria immitis* retrieved from the right atrium and ventricle. **(C, D)** Particulate materials, suspected to represent necrotic heartworm fragments, are observed adhering to the dialysis catheter and within blood filtered through gauze during catheter removal.

A 12-year-old, 7 kg, castrated male, mixed-breed dog (body condition score 4/9) presented with lethargy, vomiting, and hematuria. The dog had no significant medical history, but exhibited progressive lethargy for 2 weeks. The owner reported an absence of routine heartworm prophylaxis. Hematologic analysis revealed leukocytosis (WBC 19.1 × 10^9^/L; RI: 6–17), anemia (hematocrit 31.5%; RI: 39–56), and thrombocytopenia (platelets 59 × 10^9^/L; RI: 117–460). Serum biochemistry revealed markedly elevated levels of the following hepatic enzymes; alkaline phosphatase (ALP), 739 U/L (RI: 23–212); aspartate aminotransferase (AST), 3,867 U/L (RI: 0–50), gamma-glutamyl transferase (GGT), 6 U/L (RI: 0–11); and alanine aminotransferase (ALT), exceeding the assay's upper detection limit (RI: 10–125). Total bilirubin level was mildly elevated at 1.4 mg/dl (RI: 0.0–0.9). Kidney parameters showed increased blood urea nitrogen (BUN), 29 mg/dl (RI: 7–27) and symmetric dimethylarginine (SDMA), 23 μg/dl (RI: 0–14), whereas creatinine (CRE) remained within the normal range at 1.0 mg/dl (RI: 0–14). Inflammatory and cardiac biomarkers were markedly elevated, with C-reactive protein (CRP) at 8.3 mg/dl (RI; 0–0.1) and N-terminal pro-B-type natriuretic peptide (NT-proBNP) at 1,534.8 pmol/L (RI; 0–900). A 4Dx SNAP test (IDEXX) was positive for *Dirofilaria immitis* antigen, confirming heartworm infection. Thromboelastography performed using a viscoelastic coagulation monitor (VCM^®^, Entegrion Inc., USA) revealed a hypocoagulable profile; normal clotting time (CT), 452 s (RI; 241–470), prolonged clot formation time (CFT), 598 s (RI; 104–266), decreased α-angle, 29° (RI; 43–64), and reduced clot amplitudes (A10; 10 mm, RI; 16–30, A20; 14 mm, RI; 22–38), with significantly diminished maximum clot firmness (MCF), 18 mm (RI; 29–44), indicating impaired clot formation and reduced clot strength. Conventional coagulation parameters supported these findings, with elevated D-dimer, 2.7 μg/ml (RI; 0–0.3), prolonged activated partial thromboplastin time (aPTT), 123.4 s (RI; 75–105), and prothrombin time (PT), 22.3 s (RI; 14–19), consistent with consumptive coagulopathy and secondary fibrinolysis, suggestive of early-stage disseminated intravascular coagulation (DIC). Thoracic radiography revealed right-sided cardiomegaly, main pulmonary artery enlargement, and a mildly diffuse interstitial lung pattern (Figures 1A, D). Transthoracic echocardiography revealed numerous linear hyperechoic structures, consistent with adult *D. immitis* within the dilated right atrium, right ventricle, and main pulmonary artery, along with severe tricuspid regurgitation (Figures 2A–C). Additionally, several adult heartworms were observed within the lumen of the caudal vena cava at the level of the hepatic vein branching, showing pulsatile motion synchronized with cardiac contractions (Figure 2D). A single adult heartworm was noted at the bifurcation of the caudal vena cava, which appeared to be stationary within the vessel lumen. Clinically, the dog exhibited lethargy, weakness, and persistent hemoglobinuria suggestive of intravascular hemolysis. Persistent dark reddish-brown urine tested positive for blood on the dipstick but showed no erythrocytes on microscopy. Based on clinical signs, imaging findings, and positive antigen results, caval syndrome was diagnosed. Emergency transvenous heartworm extraction was performed via right jugular venotomy using an endoscopic retrieval basket (Model 9903112304; MTW Endoskopie, Wesel, Germany) under fluoroscopic guidance, due to the heavy worm burden and hemodynamic instability. A total of 24 adult heartworms were successfully removed in two passes from the right atrium, right ventricle, and main pulmonary artery without any intraoperative complications (Figures 3A, B). The patient was transferred to the intensive care unit for postoperative management. Follow-up echocardiography confirmed that most heartworms had been successfully removed, with a reduced tricuspid regurgitation velocity (Figures 2E–G). Within 48 h, the dog developed signs of AKI, including oliguria and elevated BUN and CRE levels. Thoracic radiography revealed pleural effusion (Figures 1B, E), and abdominal ultrasonography revealed subcapsular or perirenal fluid accumulation (Figure 2H). AKI was suspected based on oliguria and a >50% increase in serum CRE levels within 48 h, consistent with the IRIS stage II criteria. A urinary catheter was placed to ensure accurate monitoring of the urine output. Elevated urinary cystatin B levels (IDEXX) indicated renal tubular damage. Despite aggressive fluid therapy, the kidney function did not improve, necessitating PIRRT. Two sessions of PIRRT were performed leading to the gradual normalizations of kidney parameters and consistent decline in cystatin B levels. Urine output increased steadily, and the dark reddish-brown discoloration was completely resolved. Follow-up thoracic radiographs showed a reduction in cardiomegaly and improvement in pleural effusion (Figures 1C, F). The dog was discharged in stable condition after making a full clinical recovery by day 11.

## Diagnostic assessment

To prevent potential anaphylactic reactions associated with heartworm extraction, dexamethasone (0.2 mg/kg IV) and chlorpheniramine (0.4 mg/kg SC) were administered preoperatively. Cefazolin (22 mg/kg IV) and butorphanol (0.2 mg/kg IV) were administered before induction. General anesthesia was induced with propofol (4 mg/kg IV), and endotracheal intubation was performed using a 5.5 mm armored tube. Anesthesia was maintained using isoflurane delivered in oxygen via a rebreathing circuit. During anesthesia, systolic blood pressure decreased < 90 mmHg, prompting management with continuous rate infusions of dopamine (10 μg/kg/min) and dobutamine (5 μg/kg/min).

In preparation for the planned interventional heartworm extraction, coagulation assessment using thromboelastography (TEG) and standard coagulation profiles revealed a hypocoagulable state. Vitamin K (1 mg/kg SC) was administered to treat coagulopathy. Concurrently, vomiting was observed, and antiemetic therapy was initiated using metoclopramide (0.5 mg/kg IV, q12h) and maropitant (2 mg/kg SC, q24h). The prescribed oral medications included doxycycline (10 mg/kg PO, q12h), famotidine (0.5 mg/kg PO, q12h), and ursodeoxycholic acid (5 mg/kg PO, q12h). After heartworm extraction, prophylactic therapies were initiated to mitigate postoperative infections and thrombotic complications. Cefazolin (20 mg/kg IV) and dalteparin (150 IU/kg SC, q8h) were administered intravenously. Oral medications were continued, along with sildenafil (1 mg/kg PO, q12h), prednisolone (0.5 mg/kg PO, q12h), silymarin (10 mg/kg PO, q12h), and N-acetylcysteine (20 mg/kg PO, q12h). According to the American Heartworm Society (AHS) guidelines, microfilaricidal therapy with topical selamectin (12% w/v; 60 mg per dose, single application; Zoetis Inc., Parsippany, NJ, USA) was initiated before adulticidal therapy. Intravenous fluid therapy was maintained at the standard maintenance rate [(0.3 × body weight [kg]) + 70 ml/day] using Plasma Solution A (HK inno.N, Cheongju, Republic of Korea), an isotonic crystalloid supplemented with vitamin B complex, L-ornithine L-aspartate (LOLA), and sodium bicarbonate. Two days after heartworm extraction (hospitalization day 4), the body weight of the dog increased from 7 to 7.5 kg. Thoracic radiography revealed pleural effusion accompanied by decreased urine output. To accurately monitor urine production and manage fluid balance, a 6-Fr Foley catheter was inserted and intravenous fluid therapy was discontinued to prevent fluid overload. Furosemide was initially administered as a bolus (0.5 mg/kg IV), followed by a continuous rate infusion (CRI) of 0.5 mg/kg/h. Urine output was 0.85 ml/kg/h on day 4 and rose to 2.56 ml/kg/h by day 5 under furosemide CRI. The first and second PIRRT sessions on days 6 and 7 yielded urine outputs of 2.00 and 1.80 ml/kg/h, respectively. Output remained stable at 1.99 ml/kg/h on day 8, and the Foley catheter was removed on day 9. Considering the possibility of right-sided heart failure contributing to pleural effusion, sildenafil was increased to 2 mg/kg/day PO and spironolactone (2 mg/kg PO, q12h) was initiated. Despite ongoing supportive treatment, serum kidney biomarkers continued to increase until day 6. Adjunctive therapies were initiated, including phosphate binders (aluminum hydroxide 30 mg/kg, sevelamer 400 mg/dog, and lanthanum carbonate 10 mg/kg), RenaMezin^®^ (Daewoong Pharmaceutical, Seoul, Republic of Korea; 1 capsule PO, q12h), and Azodyl^®^ (1 capsule PO, q12h). Despite ongoing supportive treatment, serum kidney biomarkers continued to increase until day 6.

Due to persistent oliguria, pleural effusion, and worsening azotemia, PIRRT was initiated 4 days after heartworm extraction. PIRRT was selected because of its gradual fluid and solute removal, improved hemodynamic stability, and lower labor intensity compared with other dialysis modalities. In comparison to continuous renal replacement therapy (CRRT), PIRRT provides advantages such as lower cost, greater scheduling flexibility, and reduced nursing demands, while still maintaining better hemodynamic tolerance than conventional intermittent hemodialysis. However, its limitations include less precise fluid management and potential unsuitability for patients with severe hemodynamic instability, necessitating careful monitoring of solute clearance throughout treatment (13). Although the baseline BUN level was not markedly elevated, its rapid progression, in conjunction with pleural effusion and persistent weight gain unresponsive to diuretic therapy, indicated insufficient fluid clearance and evolving volume overload. Comprehensive clinical evaluation estimated the degree of fluid accumulation to be ~5%. Accordingly, a 4-h dialysis session was planned, taking into account the patient's hemodynamic status. The target urea reduction ratio (URR) was 45% over 4 h, calculated as [(BUN_pre – BUN_post)/BUN_pre] × 100. The Kt/V was calculated to be 0.6 using the standard Daugirdas formula, indicating modest clearance (14). To ensure hemodynamic tolerance, blood flow was initiated at 1 ml/kg/min and gradually increased to 8 ml/kg/min. Ultrafiltration was set at 8 ml/kg/h, with a total circuit blood flow of 31 ml/h. Based on the Dufayet/Cowgill method, this corresponds well to an estimated clearance of 8 ml/min for a URR of ~40% over 5–6 h (15).

PIRRT was conducted using a Prismaflex^®^ system (Baxter International Inc., Deerfield, IL, USA) in continuous venovenous hemodiafiltration (CVVHDF) mode with an HF20 dialyzer. The dialysate used was Prismasol^®^ 2 K solution. A double-lumen hemodialysis catheter (Arrow^®^, 8 Fr, 20 cm; Teleflex Inc., Wayne, PA, USA) was placed in the left jugular vein under fluoroscopic guidance, with the tip advanced to the entrance of the right atrium.

**Figure 4:**
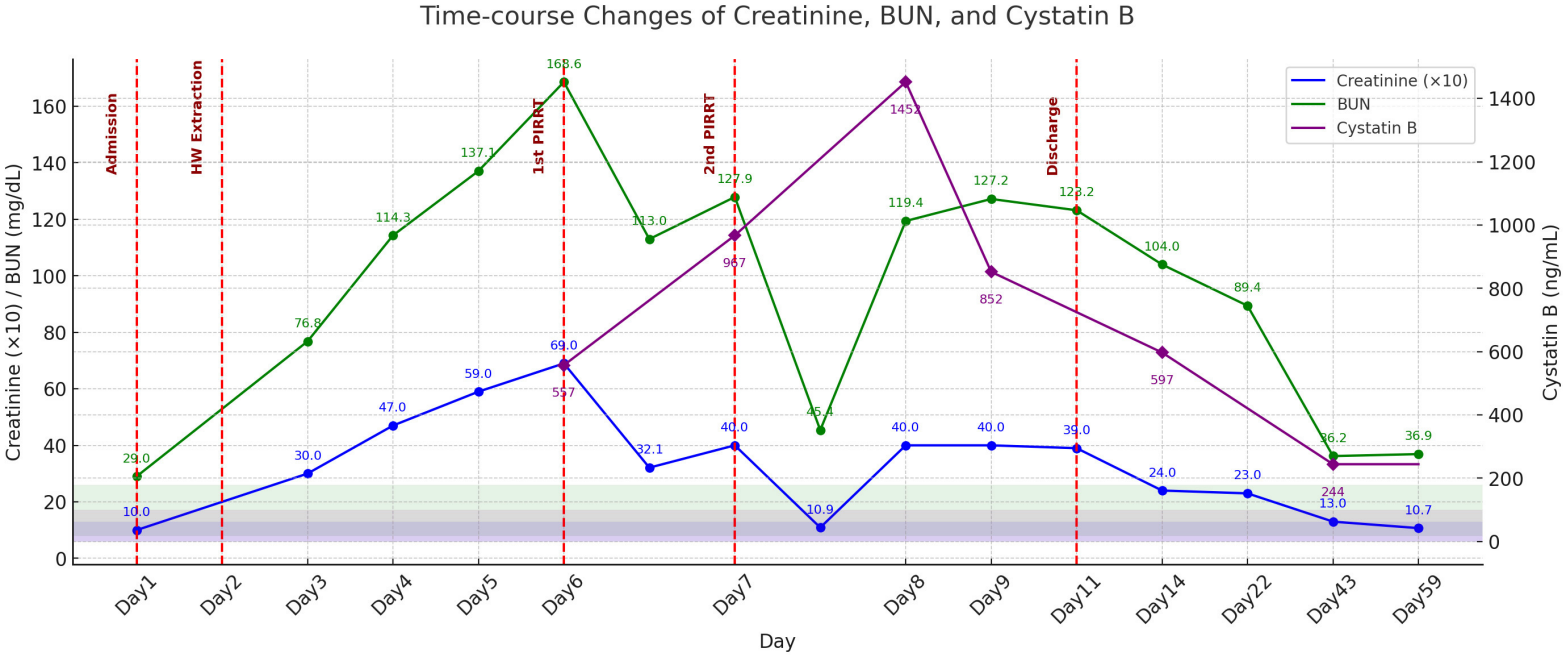
Time-course changes in serum creatinine (×10), BUN, and cystatin B concentrations during the treatment period. Notable reductions in creatinine and BUN concentrations are observed following the first and second sessions of PIRRT on days 6 and 7, respectively. Heartworm extraction is performed on day 2. Cystatin B concentrations demonstrates a gradual decline after two sessions of PIRRT. Values on day 2 are not measured; lines are linearly interpolated for continuity, with no markers shown. Shaded areas indicate normal reference ranges; key clinical interventions are marked by vertical dashed red lines. The patient was discharged on day 11.

Following heartworm extraction, hematocrit dropped to 17.2% and remained suboptimal, measuring 21.0% prior to the first PIRRT and 22.8% before the second PIRRT. To reduce hemodynamic instability and ensure adequate oxygen delivery, the extracorporeal circuit, including the HF20 hemofilter with an approximate priming volume of 60 ml, was blood-primed before each session, following confirmation of blood type and crossmatching. Anticoagulation was achieved using unfractionated heparin: the circuit was primed with 0.9% NaCl containing 2,500 IU/L heparin, followed by a 50 IU/kg bolus and continuous infusion at 25 IU/kg/h (16). PIRRT is complicated by repeated catheter occlusion requiring two replacements and filter clotting necessitating three filter changes during the two dialysis sessions. The same type of dialysis catheter was used for all placements. Catheter exchanges were performed under propofol sedation without fluoroscopic guidance. The procedure involved inserting the guidewire of the new catheter through the lumen of the existing catheter, removing the original catheter while leaving the guidewire in place, and then advancing the new dialysis catheter over the guidewire. During catheter replacement, intraluminal material presumed to represent necrotic or embolized heartworm fragments was identified, suggesting a possible role in mechanical obstruction of the dialysis system and onset of AKI (Figures 3C, D). On day 6, prior to the first PIRRT session, CRE and BUN peaked at 6.9 mg/dl and 168.6 mg/dl, respectively. After the session, CRE dropped to 3.21 mg/dl (53.5% reduction) and BUN to 113 mg/dl (33% reduction). However, rebound azotemia occurred the next day, prompting a second PIRRT session. Post-second session, CRE fell from 4.0 to 1.09 mg/dl (72.8%) and BUN from 127.9 to 45.4 mg/dl (64.5%). Following discontinuation, CRE and BUN rebounded to 3.9 and 123.2 mg/dl. Although a third session was advised, it was declined due to financial limitations. Despite this, the dog showed marked clinical improvement, regaining appetite and mobility, supporting discharge. By follow-up day 14, kidney recovery continued, with CRE at 2.4 mg/dl and BUN at 104 mg/dl, and hematocrit 27.7%. Urinary cystatin B rose from 557 ng/ml on the first dialysis day to 967 ng/ml on day two, peaking at 1,452 ng/ml after dialysis stopped. It declined to 852 ng/ml by day 8 and 597 ng/ml by day 14, paralleling clinical improvement (Figure 4). D-dimer increased to 3.0 μg/ml after heartworm extraction, then decreased to 1.2 μg/ml after the second PIRRT. By day 22, CRE was 2.3 mg/dl, BUN 89.4 mg/dl, D-dimer 0.7 μg/ml, and hematocrit 36.1%. On day 43, further improvements were noted, with CRE at 1.3 mg/dl, BUN 36.2 mg/dl, D-dimer 0.2 μg/ml, Cystatin B 244 ng/ml, ALT 588.6 U/L, and AST 39.2 U/L. At the most recent follow-up on day 59, kidney and liver parameters continued to improve, with CRE at 1.07 mg/dl, BUN 36.9 mg/dl, ALT 458 U/L, and AST 28 U/L.

## Discussion

Caval syndrome, also known as dirofilarial hemoglobinuria, is a severe and frequently fatal complication of heartworm disease (1). Although relatively uncommon, caval syndrome affects ~13%−20% of dogs with *Dirofilaria immitis* and is associated with high mortality if not managed promptly (1–3). A previous retrospective study involving 42 dogs demonstrated a survival rate of 67% at discharge following successful transvenous heartworm extraction, with >50% of these patients surviving beyond 2 years, highlighting the potential for favorable long-term outcomes with timely intervention (2). Currently, the AHS recommends a three-dose melarsomine protocol for adulticide treatment following the administration of macrocyclic lactones and doxycycline. However, in dogs presenting with significant hemodynamic compromise, the AHS guidelines support emergency surgical or transvenous extraction to prevent rapid clinical deterioration (17). Accordingly, transvenous heartworm extraction was successfully performed in this case, achieving substantial removal of adult worms and subsequent improvement in hemodynamic parameters. Despite this initial success, the patient developed AKI shortly thereafter. Although the patient did not exhibit overt hypotension or severe oliguria preoperatively, the temporal relationship strongly suggests that procedural complications were potential causative factors. Multiple pathogenic mechanisms may contribute to AKI development, including subclinical hemodynamic compromise (1, 6–12), pigment nephropathy secondary to intravascular hemolysis and hemoglobinuria (18, 19), direct renal tubular injury (20), and systemic inflammation (21, 22). Considering the rapid increase in kidney biomarkers, concurrent oliguria, and onset of pleural effusion immediately after extraction, we hypothesized that embolization of necrotic or fragmented heartworm debris during extraction was the most likely triggering event. This hypothesis is further supported by the identification of intraluminal debris consistent with fragmented worm material during catheter exchange, indicating mechanical or antigenic insults to the renal tubules, resulting in an acute inflammatory response, tubular injury, and functional impairment. Therefore, while effective heartworm removal is a key procedural goal, careful technique is equally essential to prevent renal complications. In this case, extraction was performed with deliberate attention to minimizing fragmentation and embolization risks. Although some degree of fragmentation occurred during retrieval from the endoscopic basket, the device was thoroughly flushed with sterile saline prior to reintroduction into the jugular vein. Moreover, a marked postoperative increase in urinary cystatin B, a sensitive tubular injury biomarker, provides further support for acute renal tubular damage. As a structural marker released from epithelial cells undergoing apoptosis or necrosis, cystatin B is particularly valuable for the early detection of AKI, especially during the initial phases if conventional functional biomarkers, such as serum CRE or SDMA, may remain within reference intervals (23). Although renal histopathologic evaluation was not available in this case and pre-intervention urinary cystatin B levels were not measured while serum creatinine remained within normal limits, the substantial postoperative increase in urinary cystatin B strongly supports the presence of acute tubular injury. Integrating cystatin B measurements with conventional biomarkers may enhance diagnostic precision and guide clinical decision-making in similar scenarios. In addition to cystatin B, erythropoietin (EPO) has been proposed as a potential biomarker and therapeutic agent in AKI, owing to its ability to mitigate oxidative stress and apoptosis (24). EPO has been shown to suppress tubular epithelial cell death, promote regenerative proliferation, and accelerate kidney functional recovery, thus offering further promise in the management of AKI (25).

Another contributor to AKI is the systemic inflammatory response induced by exposure to parasitic antigens. The administration of selamectin for microfilaricidal treatment concurrently with heartworm extraction may accelerate microfilarial death and antigen release. Although macrocyclic lactones are indirectly nephrotoxic, they have been reported to amplify the inflammatory cascades (21, 22). However, recent studies have indicated that kidney biomarkers typically remain stable during standard adulticidal protocols (26). Thus, although the role of selamectin cannot be entirely discounted, the severity, rapid onset, and temporal proximity of AKI in this case strongly implicate procedural factors rather than pharmacological causes. Management of AKI in this case through PIRRT poses unique clinical challenges. Despite documented systemic hypocoagulability suggestive of early disseminated intravascular coagulation, repeated episodes of filter and catheter clotting occurred. This paradox may be explained by localized coagulation pathway activation upon exposure to extracorporeal circuits, particularly in the presence of pro-inflammatory mediators, such as parasite antigens or circulating immune complexes. The extracorporeal circuit was continuously monitored to detect early signs of clotting, including visual cues such as darkened blood, fibrin deposits, or streaking, as well as pressure changes and coagulation parameters. Upon suspicion of clot formation, the anticoagulation regimen was promptly modified, incorporating targeted intra-circuit heparin boluses. Intensive anticoagulation therapy using heparin along with frequent circuit management ultimately facilitated the successful completion of PIRRT sessions.

PIRRT enables gradual and controlled solute removal while preserving hemodynamic stability, which is crucial for managing critically ill veterinary patients. Although rebound azotemia occurred after dialysis sessions, the patient exhibited marked clinical improvements, including resolution of hemoglobinuria and normalization of urine output. Cystatin B has proven valuable as an early and sensitive biomarker for renal tubular injury, enabling prompt detection and longitudinal monitoring of kidney recovery. Follow-up assessments demonstrated sustained improvement in kidney parameters and stabilization of the patient's overall clinical condition.

Collectively, this case underscores the necessity of recognizing and proactively managing kidney complications after heartworm extraction. This supports the implementation of extracorporeal kidney support modalities, such as PIRRT in dogs with severe AKI postoperatively. Future studies should elucidate the kidney effects of heartworm antigenemia, optimize the timing of antiparasitic therapies, and refine anticoagulation protocols during dialysis in heartworm-infected dogs. Knowingly, this is the first reported case documenting successful kidney recovery with PIRRT in a dog with caval syndrome complicated by AKI secondary to transvenous heartworm extraction. These findings emphasize the importance of anticipating kidney complications and integrating PIRRT into a comprehensive therapeutic approach for similar clinical presentations.

## Data Availability

The original contributions presented in the study are included in the article/supplementary material, further inquiries can be directed to the corresponding author.
